# Cardiovascular Health of U.S. Early Adolescents Assessed by the Life’s Essential 8 Metric

**DOI:** 10.1016/j.focus.2026.100497

**Published:** 2026-03-28

**Authors:** Jason M. Nagata, Christiane K. Helmer, Isaac Frimpong, Alexander W. Heuer, Keira Beltran Murillo, Nathan D. Nguyen, Oliver H. Huang, Abubakr A. Al-shoaibi, Kelley Pettee Gabriel, Erin E. Dooley, Fiona C. Baker, Holly C. Gooding

**Affiliations:** 1 Department of Pediatrics, University of California, San Francisco, San Francisco, California; 2 Department of Epidemiology, University of Alabama at Birmingham, Birmingham, Alabama; 3 Center for Health Sciences, SRI International, Menlo Park, California; 4 Division of General Pediatrics and Adolescent Medicine, Department of Pediatrics, Emory University School of Medicine, Atlanta, Georgia

**Keywords:** Teen, youth, cardiometabolic, prevention, lifestyle

## Abstract

**Introduction:**

This study aimed to characterize cardiovascular health among U.S. early adolescents using the American Heart Association’s Life’s Essential 8 metric in a large national cohort.

**Methods:**

A cross-sectional analysis of children aged 10–13 years in the Adolescent Brain Cognitive Development Study was conducted, primarily using data from the Year 2 follow-up (2018–2020), with supplemental Year 3 (2019–2021) data to improve completeness of cardiometabolic measures. Life’s Essential 8 behavioral factors scores (diet, physical activity, nicotine exposure, sleep) and health factors scores (BMI, blood pressure, blood glucose, blood lipids) as well as overall Life’s Essential 8 scores were calculated. Descriptive statistics were generated, and sex (assigned at birth) differences were assessed using Mann–Whitney *U* tests.

**Results:**

Among participants with complete Life’s Essential 8 data (*n*=1,235; mean age 12.0±0.7 years), the mean overall Life’s Essential 8 score was 78.1±10.4. Most adolescents had moderate (52.9%) or high (46.4%) cardiovascular health, whereas very few had low (0.4%) or optimal (0.3%) cardiovascular health. The mean behavioral factors score was 66.8±15.9, and the mean health factors score was 88.1±11.8. Overall Life’s Essential 8 scores did not significantly differ by sex; however, males had higher (better) physical activity and BMI scores but lower (worse) diet, nicotine exposure, sleep, and blood pressure scores than females.

**Conclusions:**

In this national sample of early adolescents, fewer than half demonstrated high cardiovascular health on the basis of Life’s Essential 8, and optimal cardiovascular health was rare. Poor diet and low physical activity were prominent contributors to suboptimal cardiovascular health. These findings highlight early adolescence as a critical window for interventions to improve long-term cardiovascular health.

## HIGHLIGHTS

• Mean overall Life’s Essential 8 score was 78.1 in U.S. adolescents.
• Most had moderate or high cardiovascular health (CVH); optimal CVH was rare.
• Diet and physical activity were key drivers of lower CVH.

## Graphical Abstract

**Figure:**
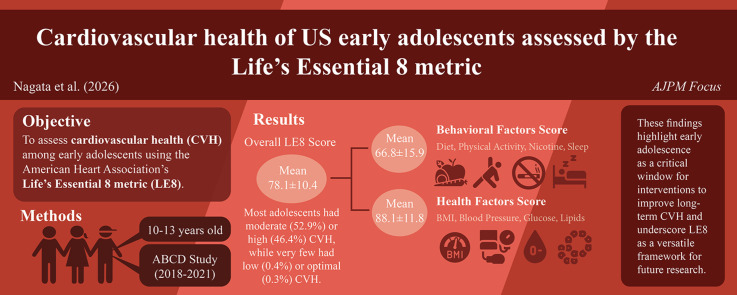

## INTRODUCTION

Cardiovascular health (CVH) is a key predictor of long-term health outcomes but remains suboptimal among U.S. children, with declines beginning in childhood and early adolescence.^1^^,^^2^ In 2022, the American Heart Association updated its CVH framework from Life’s Simple 7 to Life’s Essential 8 (LE8), introducing sleep health as the 8th metric.^1^ LE8 has demonstrated strong predictive value for adverse outcomes across the life course,^3^ including cardiovascular disease events, such as stroke and coronary heart disease,^4^ as well as both all-cause and cardiovascular mortality.^5^ Although a recent study applied LE8 across developmental stages in 2 cohort studies in Colorado and Massachussetts,^6^ data characterizing early adolescent CVH from national samples are limited.^1^ To address this gap, this study describes CVH using LE8 in the Adolescent Brain Cognitive Development (ABCD) Study, the largest longitudinal cohort study of child health and development among a demographically diverse sample of U.S. adolescents.^7^

## METHODS

### Study Population

The ABCD Study recruited 11,875 children at baseline (2016–2018) using a multistage probability sampling framework implemented across 21 sites in the U.S. to approximate the sociodemographic composition of the U.S. population.^8^ Cross-sectional data came from early adolescents aged 10–13 years, primarily from the Year 2 follow-up (2018–2020) of the ABCD Study. Behavioral factors scores (average of diet, physical activity, nicotine exposure, and sleep scores) were calculated among participants with complete data for all 4 behavioral components, and health factors scores (average of BMI, blood pressure, blood glucose, and blood lipids scores) were calculated among participants with complete data for all 4 health components.^1^ Among participants with complete data for all 8 components, the overall LE8 CVH score was calculated as the average of all 8 components.^1^ Details on the scoring of each component are provided subsequently and in Table 1. Of the 10,885 participants who had data for at least 1 LE8 component, 1,235 participants had complete data for all 8 components. Sociodemographic differences between those with and without complete LE8 data are found in Appendix A (available online).

**Table 1:** Definition and Scoring Approach for Quantifying Cardiovascular Health Based on the American Heart Association’s Life's Essential Eight (LE8), as Implemented in the National Health and Nutrition Examination Survey (NHANES, Lloyd-Jones et al.^1^) and Applied in the Adolescent Brain Cognitive Development Study for the Present Analysis

LE8 component	Lloyd-Jones et al.^1^ quantification of CVH metric, children (up to age 19 years)	Nagata et al. quantification of CVH metric, early adolescents (aged 10–13 years)
Diet	Measurement recommendation: Self-reported daily intake of a DASH-style eating pattern using NHANES DASH diet score Quantiles of DASH-style diet adherence; ages 2–19 Scoring (population): Points quantile 100 ≥95th %ile (top/ideal diet) 80 75th–94th %ile 50 50th–74th %ile 25 25th–49th %ile 0 1st–24th %ile (bottom/least ideal quartile)	Measurement: DASH-style diet calculated from parent-reported past-week Block Kids Food Screener Quantiles of DASH-style diet adherence; ages 10–13 scoring (within ABCD): Points quantile 100 ≥95th %ile (top/ideal diet) 80 75th–94th %ile 50 50th–74th %ile 25 25th–49th %ile 0 1st–24th %ile (bottom/least ideal quartile)
Physical activity	Measurement recommendation: Self-reported minutes of moderate or vigorous physical activity per week from NHANES PAQ-K questionnaire Metric: Minutes of moderate- (or greater) intensity activity per week; ages 2–19 years Scoring: Points minutes 100 ≥420 90 360–419 80 300–359 60 240–299 40 120–239 20 1–119 0 0	Measurement: Parent-reported participation, frequency, and duration from Sports and Activity Involvement Questionnaire and MET assignments from Youth Compendium of Physical Activities updated by Butte et al. Metric: MET hours per week of moderate- (or greater) intensity physical activity per week; ages 10–13 years Scoring: Points MET hours per week 100 ≥21 90 18.0–20.9 80 15.0–17.9 60 12.0–14.9 40 6.0–11.9 20 >0–5.9 0 0
Nicotine	Measurement recommendation: Self-reported use of cigarettes or inhaled nicotine-delivery system from NHANES SMQ Metric: Combustible tobacco use and/or inhaled NDS use or second-hand smoke exposure; ages 12–19 Scoring: Points status 100 Never tried 50 Tried any nicotine product but >30 days ago 25 Currently using inhaled NDS 0 Current combustible use (any within 30 days) Subtract 20 points (unless score is 0) for living with active indoor smoker in home	Measurement: Self-reported ever/past use of cigarettes or inhaled NDS from substance use interview and parent-reported second-hand smoke from Survey of Substance Use Density, Storage, and Exposure Metric: Combustible tobacco use and/or inhaled NDS use or second-hand smoke exposure and exposure; ages 10–13 Scoring: Points status 100 Never tried 50 Tried any nicotine product but >30 days ago 0 Current combustible use (any within 30 days) Subtract 20 points (unless score is 0) for living with active indoor smoker in home
Sleep	Measurement recommendation: Self-reported average hours of sleep per night from *On average, how many hours of sleep do you get per night?* Consider objective sleep/actigraphy data from wearable technology, if available Metric: Average hours of sleep per night; ages 16–19 Scoring: Points level 100 Age-appropriate optimal range 90 <1 hour above optimal range 70 <1 hour below optimal range 40 1 to <2 hours below or ≥1 hour above optimal 20 2 to <3 hours below optimal range 0 ≥3 hours below optimal range	Measurement: Sleep duration calculated from self-reported sleep and wake times from the Munich Chronotype Questionnaire Metric: Average hours of sleep per night; ages 10–13 Scoring: 100 9–12 hours (<13 years), 8–10 hours (≥13 years) 90 >12–13 hours (<13 years), >10–11 hours (≥13 years) 70 8 to <9 hours (<13 years), 7 to <8 hours (≥13 years) 40 7 to <8 hours or >13 hours (<13 years), 6 to <7 hr or >11 hours (≥13 years) 20 6 to <7 hours (<13 years), 5 to <6 hours (≥13 years) 0 <6 hours (<13 years), <5 hours (≥13 years)
BMI	Measurement recommendation: Body weight (kg) divided by height squared (m^2^) from objective measurement of height and weight Metric: BMI %iles for age and sex; ages 2–19 Scoring: Points level 100 5^th^ to <85th %ile 70 85^th^ to <95th %ile 30 95th %ile to <120% of the 95th %ile 15 120% of the 95th %ile to <140% of the 95th %ile 0 ≥140% of the 95th %ile	Measurement: Body weight (kg) divided by height squared (m^2^) from objective measurement of height and weight Metric: BMI percentiles (%iles) for age and sex; ages 10–13 years Scoring: Points level 100 5th to <85th %ile 70 85th to <95th %ile 30 95th %ile to <120% of the 95th %ile 15 120% of the 95th %ile to <140% of the 95th %ile 0 ≥140% of the 95th %ile
Blood lipids	Measurement recommendation: Plasma total and HDL cholesterol with calculation of non-HDL cholesterol from fasting or nonfasting blood sample Metric: Non-HDL cholesterol (mg/dL); ages 6–19 years Scoring: Points level 100 <100 60 100–119 40 120–144 20 145–189 0 ≥190 If drug-treated level, subtract 20 points	Measurement: Plasma total and HDL cholesterol with calculation of non-HDL cholesterol from nonfasting blood sample Metric: Non-HDL cholesterol (mg/dL); ages 10–13 years Scoring: Points level 100 <100 60 100–119 40 120–144 20 145–189 0 ≥190 Of those who had lipids data in ABCD, none were using lipid-lowering drugs, so no subtracting was applied.
Blood glucose	Measurement recommendation: FBG or casual HbA1c from fasting (FBG, HbA1c) or nonfasting (HbA1c) blood sample Metric: Fasting blood glucose (mg/dL) or HbA1c (%); ages 12–19 years Scoring: Points level 100 No history of diabetes and FBG <100 (or HbA1c < 5.7) 60 No diabetes and FBG 100–125 (or HbA1c 5.7–6.4) (prediabetes) 40 Diabetes with HbA1c <7.0 30 Diabetes with HbA1c 7.0 – 7.9 20 Diabetes with HbA1c 8.0 – 8.9 10 Diabetes with Hb A1c 9.0 – 9.9 0 Diabetes with HbA1c ≥10.0	Measurement: Hemoglobin A1c from non-fasting (HbA1c) blood sample Metric: HbA1c (%) and/or parent-reported history of diabetes; ages 10–13 years Scoring: Points level 100 No history of diabetes and HbA1c < 5.7 60 No diabetes and HbA1c 5.7–6.4 (prediabetes) 40 Diabetes with HbA1c <7.0 30 Diabetes with HbA1c 7.0–7.9 20 Diabetes with HbA1c 8.0–8.9 10 Diabetes with Hb A1c 9.0–9.9 0 Diabetes with HbA1c ≥10.0
Blood pressure	Measurement recommendation: Appropriately measured SBP and DBP with blood pressure cuff Metric: SBP and DBP (mmHg) percentiles for ages 8–12 years. For ages ≥13 years, use adult scoring. Scoring: Points level 100 Optimal (<90th %ile) 75 Elevated (≥90^th^ to <95th %ile or ≥120/80 mmHg to <95th %ile, whichever is lower) 50 Stage I HTN (≥95th to <95th %ile + 12 mmHg, or 130/80 to 139/89 mm Hg, whichever is lower) 25 Stage 2 HTN (≥95th %ile + 12 mmHg or ≥140/90 mmHg, whichever is lower) 0 SBP ≥160 or ≥95th %ile + 30 mmHg systolic, whichever is lower; and/or DBP ≥100 or ≥95th %ile + 20 mmHg diastolic Subtract 20 points if treated level	Measurement: Measured SBP and DBP with blood pressure cuff Metric: SBP and DBP (mmHg) percentiles for ages 10–12 years. For ages ≥13 years, adult scoring was used. Scoring: Points level 100 Optimal (<90th %ile) 75 Elevated (≥90^th^ to <95th %ile or ≥120/80 mmHg to <95th %ile, whichever is lower) 50 Stage I HTN (≥95th to <95th %ile + 12 mmHg or 130/80 to 139/89 mmHg, whichever is lower) 25 Stage 2 HTN (≥95th %ile + 12 mmHg or ≥140/90 mmHg, whichever is lower) 0 SBP ≥160 or ≥95th %ile + 30 mmHg systolic, whichever is lower and/or DBP ≥100 or ≥95th %ile + 20 mmHg diastolic Subtract 20 points if treated level

%ile, percentile; ABCD, Adolescent Brain Cognitive Development; AHA, American Heart Association; CVH, cardiovascular health; DASH, Dietary Approaches to Stop Hypertension; DBP, diastolic blood pressure; FBG, fasting blood glucose; HDL, high-density lipoprotein; HTN, hypertension; LE8, Life’s Essential 8; MET, metabolic equivalent of task; NDS, nicotine-delivery system; NHANES, National Health and Nutrition Examination Survey; PAQ, Physical Activity Questionnaire; SMQ, Smoking Cigarette Use Questionnaire; SBP, systolic blood pressure.

### Measures

As mentioned previously, components of the LE8 were primarily assessed during the Year 2 follow-up (2018–2020). Blood samples were only collected starting in Year 2, from a portion of participants who consented to a blood draw. A blood draw was not available for almost half the sample who were assessed during the coronavirus disease 2019 (COVID-19) pandemic, owing to the suspension of nonessential research activities. During Year 3 follow-up (2019–2021), a blood draw option was offered to some participants who had missed this assessment in Year 2. Therefore, data from both Year 2 and Year 3 were combined to maximize sample completeness for cardiometabolic risk data requiring blood samples (glucose and lipids) and were available on a small subset of less than 2,000 participants. Similarly, owing to COVID-19–related restrictions during Year 2, anthropometric measurements (height and weight for BMI calculation) and blood pressure were collected from a reduced number of participants. To improve data completeness for BMI and blood pressure, measurements from both Year 2 and Year 3 were combined using the same approach. Therefore, the health factors score was primarily derived from Year 2 follow-up data and supplemented by Year 3 data for participants who missed the Year 2 follow-up.

The Block Kids Food Screener (BKFS), created by NutritionQuest, is a dietary assessment tool consisting of a 41-item food frequency questionnaire.^9^ The questionnaire estimates the nutrient intake and food group consumption of children aged 2–17 years.^9^ The BKFS demonstrated strong relative validity when compared with three 24-hour dietary recalls in the Nutrition Data System for Research, with correlations ranging from 0.5 to 0.9.^9^

The BKFS asked parents/caregivers to provide how often and how much food and drink their child consumed during the previous week. These responses were then reviewed by the child. The present analysis utilized the Year 2 BKFS data provided by NutritionQuest and included estimated average daily consumption across several food groups. These categories included ounce/cup equivalents of fruit/fruit juice, vegetables, whole grains, meat/poultry/fish, dairy, and legumes. To differentiate between fruit and fruit juice categories, the fruit juice variable was converted from gram to cup equivalents and subtracted from the fruit/fruit juice cup equivalents variable. Summary data from BKFS for micronutrients, such as milligrams of sodium and iron, were also provided. Food measures were converted from cups and ounces to grams and standardized dietary intake per 1,000 kcal (4,128 kJ), consistent with other BKFS analyses.^10^^,^^11^ To standardize across a range of overall food consumption, dietary intake of food groups and micronutrients for each participant was divided by their total caloric intake and multiplied by 1,000 kcal.

A Dietary Approaches to Stop Hypertension (DASH) score was constructed on the basis of food and nutrient intake, focusing on 8 components: high intake of fruits, vegetables, nuts and legumes, low-fat dairy products, and whole grains and low intake of sodium, sweetened beverages, and red and processed meats.^12^ For vegetables, legumes, whole grains, and sodium, standardized BKFS estimates were used. For the remaining components, the following categorizations were applied: low-fat dairy products included nonfat milk, 1% milk, and 2% milk; red and processed meats included hamburgers or cheeseburgers, hot dogs or corn dogs, lunch meats, beef such as roast, steak, meatballs, and meatloaf, and pork such as chops and roast; and sweetened beverages included soft drinks and other sweetened drinks (e.g., Coke, Sunny Delight), chocolate milk, and fruit juice.

For each of the 8 DASH components, participants were assigned to an intake quintile on the basis of the distribution within the ABCD cohort. Higher quintiles reflected greater intake for components considered healthy (e.g., fruits, vegetables), whereas lower intake of components considered unhealthy (e.g., sodium, sweetened beverages) was reverse coded so that higher quintiles still reflected healthier consumption.^12^ Each participant’s DASH score was calculated by summing the assigned quintile values across all 8 components, resulting in a possible range of 8 to 40, with higher scores indicating greater adherence to a DASH-style diet. After calculating DASH scores, each participant was assigned a percentile ranking on the basis of the distribution of DASH scores within the ABCD cohort. This internal percentile classification allowed assessment of relative adherence to the DASH-style diet while addressing 2 limitations: potential underreporting in BKFS dietary estimates and the lack of an appropriate external reference group for comparison.

Regarding physical activity, the Sports Activity Involvement Questionnaire (SAI-Q) assesses past-year (since previous assessment) participation in 23 different sports as well as extracurricular activities and hobbies such as music and drawing. The SAI-Q was adapted from the Vermont Health and Behavioral Questionnaire and the Dutch Health Behavior Questionnaire.^13^

Parents/caregivers were asked to provide (1) duration of time in minutes spent per session (duration per session), (2) frequency of participation per week (days per week), and (3) number of months participating per year (months per year) for each sport/activity. Each sport and extracurricular activity was assigned an age-specific metabolic equivalent of task (MET) value ranging from 0 to 10 (none to highest intensity). MET values were determined using the Youth Compendium of Physical Activities.^14^^,^^15^ Intermediate and summary scores in MET hours per week were calculated using standard equations on the basis of reported duration, frequency, and assigned intensity by reported activity type as further described by Nagata et al.^16^ For this analysis, minute per week thresholds provided by Lloyd-Jones and colleagues^1^ were converted to MET hours per week using the following calculation: ([minutes per week × 3 METs, i.e., lower threshold for moderate intensity physical activity]/60 minutes).

Nicotine exposure was assessed using adolescent and parent/caregiver surveys.^17^ Adolescents who responded *yes* to, *Have you ever tried a puff from a tobacco or electronic cigarette, or vape pens, or e-hookah at any time in your life?* and/or *Have you ever tried nicotine replacement, such as patches, gums, nasal sprays, inhalers, and lozenges at any time in your life?* were coded as having ever used nicotine. Participants were also asked how many days ago they last used tobacco cigarettes, e-cigarettes, or nicotine replacement products. However, the responses to these questions were not available, preventing classification of current users (within 30 days). Therefore, participants could only be classified as ever users and could not be identified as current users.

In addition, parents completed the ABCD Parent Survey of Substance Use Density, Storage, and Exposure.^17^ They were asked, *Did anyone smoke cigarettes inside the house when your child was home or in a vehicle your child was in?* An affirmative response was coded as exposure to second-hand smoke.

Adolescent-reported sleep duration was assessed using the 17-item Munich Chronotype Questionnaire, which collects self-reported information on bedtimes and wake times for both school days and free days.^18^ Weekdays were labeled as school days, and weekends or nonschool days were labeled as free days.^19^ Average weekly sleep duration was derived using a weighted formula: ([school day sleep duration × school days per week] + [free day sleep duration × free days per week])/7.^19^ The American Academy of Sleep Medicine’s sleep recommendations were utilized to evaluate adequate sleep in adolescents with recommended sleep duration as 9–12 hours a night for those aged 6–12 years and 8–10 hours for those aged 13–18 years.^20^

Weight was assessed using a high-capacity digital scale, and height was measured with a tape measure by trained research assistants. An average of 3 measurements for height and weight was recorded. Average height and weight values were converted into BMI percentiles for age and sex, following guidelines from the Centers for Disease Control and Prevention growth charts.^21^

Regarding blood lipids, nonfasting blood samples were collected using standard venipuncture methods and processed according to established laboratory procedures.^22^ Non–high-density lipoprotein cholesterol was calculated by subtracting high-density lipoprotein cholesterol from the total cholesterol.

Blood glucose was assessed by measuring hemoglobin A1c. Adolescents with a history of diabetes (parent reported) were also included in the scoring criteria.

Each participant’s blood pressure was measured 3 times consecutively with an automatic sphygmomanometer, with 1-minute intervals between each measurement. The average of the 3 measurements was used for analysis. Average systolic and diastolic values were then converted into age- and sex-specific percentiles on the basis of American Academy of Pediatrics guidelines.^23^ Participants were then classified as having either normal or hypertensive-range (high) blood pressure.^24^

### Statistical Analysis

Data analyses were performed in Stata 18 (StataCorp, College Station, TX). Descriptive statistics were calculated using a complete-case approach and summarized with means, SDs, medians, and IQRs. Analyses stratified by sex assigned at birth were conducted using 2-sample Wilcoxon rank-sum (Mann–Whitney *U*) tests. IRB approval was obtained from the University of California, San Diego as well as each of the 21 participating research sites. Caregivers provided written consent, and adolescents provided written assent.

## RESULTS

Of the ABCD participants who had data for at least 1 LE8 factor (*n*=10,885), the average age at the 2-year follow-up was 12.0±0.7 years. The sample was racially and ethnically diverse, with 46.3% identifying as minorities (6.1% Asian, 19.3% Black, 16.6% Latino/Hispanic, 3.4% Native American, 0.9% other). Household income was below $75,000 for 39.5% of families and $75,000 or more for 60.5%. Most parents (90.0%) held a college degree or higher; 10.0% had a high-school education or less. Participants with missing data were statistically more likely to be from racial and ethnic minority groups and lower-income households than those who had complete data available for an LE8 score (*n*=1,235) (Appendix A, available online).

Table 2 shows the overall LE8 scores and contributing factor scores for male and female participants. Of those with complete LE8 data (*n*=1,235), the overall LE8 score was 78.1±10.4. Most participants had moderate (52.9%) or high (46.4%) scores, with very few in the low or optimal categories (0.4% and 0.3%, respectively). The behavioral factors score (*n*=9,151) was 66.8±15.9, and the health factors score (*n*=1,356) was 88.1±11.8. There was no statistically significant difference in overall LE8 scores by sex, but males scored higher (better) than females in physical activity and BMI but lower (worse) in diet, nicotine exposure, sleep, and blood pressure (Appendix B, available online, and Table 2).

**Table 2:** Life's Essential 8 Cardiovascular Health Characteristics of Participants Aged 10–13 Years in the Adolescent Brain Cognitive Development Study at Year 2 (2018–2020), Overall and Stratified by Sex

Characteristics	Mean±SD/*n* (%) CVH score		
	Overall	Female	Male
Overall CVH summary score (*n*=1,235)	78.1±10.4	77.6±10.2	78.5±10.6
Low (0 to <50 points)	5 (0.4%)	1 (0.2%)	4 (0.6%)
Moderate (50 to <80 points)	653 (52.9%)	317 (56.1%)	336 (50.1%)
High (80 to <100 points)	573 (46.4%)	246 (43.5%)	327 (49.2%)
Optimal (100 points)	4 (0.3%)	1 (0.2%)	3 (0.7%)
Behavioral factors subcomponent score (*n*=9,151)	66.8±15.9	67.0±15.9	66.7±15.9
Low (0 to <50 points)	1,186 (13.0%)	563 (13.0%)	623 (13.0%)
Moderate (50 to <80 points)	5,618 (61.4%)	2,685 (61.9%)	2,929 (60.9%)
High (80 to <100 points)	2,263 (24.7%)	1,044 (24.1%)	1,219 (25.4%)
Optimal (100 points)	84 (0.9%)	48 (1.1%)	36 (0.7%)
Behavioral factors			
Diet (*n*=10,885)	37.1±31.0	40.4±31.5	34.1±30.2
Nicotine exposure (*n*=10,002)	98.4±8.01	98.7±7.2	98.2±8.7
Physical activity (*n*=10,206)	42.6±41.1	38.9±40.2	46.1±41.5
Sleep duration (*n*=10,381)	86.2±23.2	86.7±22.7	85.7±23.7
Health factors subcomponent score^a^ (*n*=1,356)	88.1±11.8	87.4±11.9	88.7±11.6
Low (0 to <50 points)	5 (0.4%)	3 (0.5%)	2 (0.3%)
Moderate (50 to <80 points)	273 (20.1%)	135 (21.8%)	138 (18.7%)
High (80 to <100 points)	610 (45.0%)	287 (46.4%)	323 (43.8%)
Optimal (100 points)	468 (34.5%)	194 (31.3%)	274 (37.2%)
Health factors^a^			
BMI (*n*=7,746)	80.9±29.7	78.7±30.5	82.8±28.8
Blood glucose (*n*=1,607)	98.6±8.5	98.6±8.2	98.7±8.7
Blood lipids (*n*=1,475)	75.8±27.3	75.0±27.9	76.4±26.8
Blood pressure (*n*=5,995)	96.8±11.1	97.3±10.2	96.3±11.7

*Note*: All CVH scores range from 0 to 100, with 100 representing optimal health.

a Owing to in-person restrictions during the COVID-19 pandemic, health factor subcomponent scores were primarily derived from Year 2 follow-up data, supplemented by Year 3 data for participants who missed the Year 2 follow-up. ABCD, Adolescent Brain Cognitive Development; CVH, cardiovascular health.

## DISCUSSION

Less than half of ABCD participants aged 10–13 years had high overall CVH on the basis of LE8, with only 0.3% reaching an optimal LE8 score of 100 points. The average overall LE8 score was moderate, with poor diet and physical activity scores. Compared with those aged 11–16 years from the ECHO Consortium (Healthy Start in Colorado and Project Viva in Massachusetts), overall LE8 scores were similar^6^; however, the ABCD cohort had lower physical activity and blood pressure scores and higher BMI scores. Although males had lower overall LE8 scores than females in the ECHO Consortium,^6^ there was no overall difference in total LE8 score by sex in this study. However, male adolescents in the ABCD sample scored higher in physical activity and BMI and lower in diet, nicotine exposure, sleep, and blood pressure than female adolescents. Compared with adolescents in the Lloyd-Jones et al.’s^1^ study, which analyzed National Health and Nutrition Examination Survey (NHANES) data from U.S. adolescents (of varying age ranges depending on LE8 component data availability) between 2013 and 2018 (before COVID-19), adolescents in the present study had slightly higher overall LE8 scores, with higher subscores in nicotine exposure, sleep, BMI, and blood glucose but lower scores in physical activity and blood pressure.

It is important to note that these comparisons should be interpreted with caution because the age ranges and COVID-19 pandemic timing were different. In addition, the operationalization of individual LE8 components differed between ABCD and the reference studies owing to data availability. For example, on the basis of what measures were available in ABCD, the operationalization for this study was adapted from Lloyd-Jones and colleagues’^1^ definitions, which used NHANES. In ABCD, diet was scored by converting BKFS responses into a DASH-style adherence score with within-sample percentiles, and physical activity was quantified as MET hours per week using the SAI-Q. Although the instruments and thresholds differ for these components, the approaches used were conceptually similar to those used in the NHANES study, and differences in scoring for the remaining components were relatively minor. In addition, compared with the Healthy Start and Project Viva cohorts,^6^ which also adapted their operationalization from Lloyd-Jones et al.,^1^ the primary difference was that this study used DASH-style adherence scores rather than the Healthy Eating Index 2015, which may have partially explained the observed discrepancies.

Improving LE8 scores in adolescence is critical because they are inversely associated with cardiovascular disease risk across the lifecourse.^3^ The findings of this study highlight diet and physical activity as key early behavioral intervention targets among adolescents, as supported by the American Heart Association’s 2025 Heart Disease and Stroke Statistics.^25^ School-based interventions can improve adolescent CVH,^26^ and nutrition education can benefit CVH into adulthood.^27^ Physicians should continue to monitor adolescent blood pressure, glucose, and lipids and intervene per guidelines when warranted.^23^

### Limitations

Limitations to this study include the use of self-reported behavioral data, with diet, sleep, and nicotine exposure potentially subject to social desirability and recall bias. Low physical activity may be attributed to the questionnaire’s focus on sports and extracurricular activities, potentially overlooking other forms of physical activity, and the fact that half of the sample was surveyed during COVID-19 restrictions. In addition, disruptions in cardiometabolic and anthropometric data collection during COVID-19 resulted in a smaller LE8 sample. Furthermore, only 1,235 of the 10,885 participants with data for at least 1 LE8 component had complete LE8 data. Given that participants with missing data were more likely to identify as members of racial and ethnic minority groups and to come from lower-income households, the generalizability of these findings may be limited. In addition, although the ABCD Study was designed to minimize selection bias and approximate national representation, alignment with U.S. population distributions for race, sex, and SES may vary across outcomes.^28^ Finally, survey weights were not applied because survey weights are not available for the Year 2 or 3 follow-up sample, and 2-sample Wilcoxon rank-sum (Mann–Whitney *U*) tests do not accommodate complex survey weights; thus, results may not be fully population representative.

## CONCLUSIONS

Nonetheless, this study characterizes LE8 in a large national cohort of adolescents, providing a foundation for future adolescent CVH research, especially within the ABCD Study. In future ABCD studies, LE8 can be leveraged both as an outcome and as a predictor. As an outcome, it may help elucidate how social determinants, environmental factors, and behavioral exposures influence CVH trajectories across adolescence. Conversely, as a predictor, LE8 can be used to examine the downstream impact of CVH on cardiometabolic risk, cognitive and brain development, mental health, and overall physical functioning. These considerations highlight LE8 as a versatile framework for future research within the ABCD Study to better understand and promote CVH throughout adolescence.

## Appendix Supplementary materials

Image, application 1

## Footnotes

Supplementary material associated with this article can be found in the online version at doi:10.1016/j.focus.2026.100497.
