# Supplementary material for: Cardiovascular Health of U.S. Early Adolescents Assessed by the Life’s Essential 8 Metric
Source: AJPM Focus. 2026 Mar 28;5(5):100497. doi: 10.1016/j.focus.2026.100497 (PMC13487616; doi:10.1016/j.focus.2026.100497)
Supplement: Supplementary file 1 [file mmc1.docx]

Supplementary Material

Cardiovascular health of US early adolescents assessed by the Life’s Essential 8 metric

Jason M. Nagata, MD, MSc, Christiane K. Helmer, MPH, Isaac Frimpong, MD, MPH, Alexander Heuer, Keira Beltran Murillo, BS, Nathan D. Nguyen, BS, Oliver Huang, Abubakr A. Al-shoaibi, PhD, Kelley Pettee Gabriel, PhD, Erin Dooley, PhD, Fiona C. Baker, PhD, Holly C. Gooding, MD

*American Journal of Preventive Medicine Focus*

| Appendix A. Comparison of the sociodemographic characteristics of the Adolescent Brain Cognitive Development (ABCD) Study participants with complete Life’s Essential 8 (LE8) data vs. incomplete LE8 data | | | |
| --- | --- | --- | --- |
| Sociodemographic characteristics | Participants with complete LE8 data  (n=1,235) | Participants with incomplete LE8 data (n=10,727) | p |
| Age (years) | 12.9 (0.7) | 12.0 (0.7) | 0.053 |
| Sex |  |  | 0.202 |
| Female | 45.6% | 52.4% |  |
| Male | 54.4% | 47.6% |  |
| Race and ethnicity |  |  | **<0.001** |
| Asian | 4.4% | 6.2% |  |
| Black | 12.9% | 21.1% |  |
| Latino/Hispanic | 13.4% | 17.6% |  |
| Native American | 4.0% | 3.4% |  |
| Other | 0.8% | 0.9% |  |
| White | 64.4% | 50.8% |  |
| Household income |  |  | **<0.001** |
| $24,999 or less | 10.9% | 14.1% |  |
| $25,000 to $49,999 | 10.9% | 14.0% |  |
| $50,000 to $74,999 | 14.2% | 13.0% |  |
| $75,000 to $99,999 | 15.0% | 13.1% |  |
| $100,000 to $199,999 | 35.7% | 32.2% |  |
| $200,000 or greater | 13.3% | 13.7% |  |
| Parent's highest education |  |  | 0.574 |
| High school education or less | 9.0% | 9.5% |  |
| College education or more | 91.0% | 90.5% |  |

| Appendix B. Life's Essential 8 cardiovascular health (CVH) characteristics of 10–13-year-old participants in the Adolescent Brain Cognitive Development (ABCD) Study at Year 2 (2018–2020), overall and stratified by sex | | | | |
| --- | --- | --- | --- | --- |
|  | Median (IQR) CVH score | | |  |
|  | Overall | Female | Male | p-value |
| Overall CVH Summary Score (N=1,235) | 78.1 (71.3–86.3) | 78.1 (71.3–85.0) | 79.4 (71.9–86.3) | 0.076 |
| Behavioral Factors Subcomponent Score (N=9,151) | 67.5 (55.0–80.0) | 67.5 (56.3–87.5) | 67.5 (55.0–80.0) | 0.776 |
| Diet (N=10,885) | 25.0 (0.0–50.0) | 25.0 (25.0–80.0) | 25.0 (0.0–50.0) | **<0.001** |
| Nicotine exposure (N=10,002) | 100.0 (100.0–100.0) | 100.0 (100.0–100.0) | 100.0 (100.0–100.0) | **0.012** |
| Physical activity (N=10,206) | 40.0 (0.0–90.0) | 20.0 (0.0–90.0) | 40.0 (0.0–100.0) | **<0.001** |
| Sleep duration (N=10,381) | 100.0 (70.0–100.0) | 100.0 (70.0–100.0) | 100.0 (70.0–100.0) | **0.041** |
| Health Factors Subcomponent Score^a^ (N=1,356) | 90.0 (82.5–100.0) | 90.0 (80.0–100.0) | 90.0 (82.5–100.0) | **0.029** |
| Body mass index (N=7,746) | 100.0 (70.0–100.0) | 100.0 (70.0–100.0) | 100.0 (70.0–100.0) | **<0.001** |
| Blood glucose (N=1,607) | 100.0 (100.0–100.0) | 100.0 (100.0–100.0) | 100.0 (100.0–100.0) | 0.674 |
| Blood lipids (N=1,475) | 100.0 (60.0–100.0) | 100.0 (60.0–100.0) | 100.0 (60.0–100.0) | 0.386 |
| Blood pressure (N=5,995) | 100.0 (100.0–100.0) | 100.0 (100.0–100.0) | 100.0 (100.0–100.0) | **<0.001** |
| All CVH scores range from 0-100, with 100 representing optimal health. P-values calculated using two-sample Wilcoxon rank-sum (Mann-Whitney U) tests comparing scores by sex. **Bold** indicates statistical significance defined as p<0.05. | | | | |
| ^a^Due to in-person restrictions during the COVID-19 pandemic, health factor subcomponent scores were primarily derived from Year 2 follow-up data, supplemented by Year 3 data for participants who missed the Year 2 follow-up. | | | | |
